# Loka: A Cross-Platform Virtual Reality Streaming Framework for the Metaverse

**DOI:** 10.3390/s25041066

**Published:** 2025-02-11

**Authors:** Hsiao-Wen Kao, Yan-Cyuan Chen, Eric Hsiao-Kuang Wu, Shih-Ching Yeh, Shih-Chun Kao

**Affiliations:** 1 Department of Computer Science and Information Engineering, National Central University, Taoyuan 320317, Taiwan; datting@cht.com.tw (H.-W.K.); hsiao@csie.ncu.edu.tw (E.H.-K.W.); shihching.yeh@g.ncu.edu.tw (S.-C.Y.); 2 Department of Planning, ChungHwa Telecom Laboratories, Taoyuan 326402, Taiwan; 3 Department of Health and Kinesiology, Purdue University, West Lafayette, IN 47907, USA; kao28@purdue.edu

**Keywords:** Metaverse, virtual reality, cloud rendering, internet of things

## Abstract

As the concept of the Metaverse evolves, virtual reality (VR) plays a pivotal role in creating immersive, socially interactive environments that form the backbone of this interconnected digital universe. However, VR technology often faces significant challenges, including hardware limitations, platform incompatibilities, and difficulties supporting seamless multiplayer experiences. VR streaming offers a potential solution by offloading computational tasks to remote servers, enabling high-quality VR experiences on lower-end devices and enhancing accessibility to a broader audience. In this paper, we present Loka, a versatile and extensible VR streaming framework designed to address these challenges, providing the necessary infrastructure to support social interactions and real-time collaboration in virtual environments—the key components of the Metaverse. Loka is built on Unity Engine and WebRTC, enabling seamless cross-platform VR experiences without the need for device-specific SDKs. It also supports real-time integration of custom sensory data streams, such as motion capture and physiological signals from IoT devices, which can enhance user interaction and personalization in virtual environments, as well as provide a more convenient accessible platform for research. Furthermore, Loka’s native multiplayer and multicasting capabilities facilitate collaborative and interactive social experiences, aligning with the core goals of the Metaverse. By leveraging cloud-based rendering with low-latency streaming, Loka allows users to engage in immersive VR environments on a wide range of devices, without requiring high-end hardware. Its modular architecture ensures extensibility, allowing researchers and developers to integrate new data types and experimental setups more easily. With its ability to set up immersive VR scenes to support social interaction and handle complex virtual environments, we believe the proposed work can be leveraged to foster the development and research of the Metaverse.

## 1. Introduction

The emergence of virtual reality (VR) technology has fundamentally transformed the way users engage with digital content and facilitated the creation of immersive environments that blur the boundaries between the physical and virtual realms. VR enables users to interact with three-dimensional, computer-generated spaces in real time, providing a level of engagement far beyond that of traditional two-dimensional interfaces. Although VR initially gained popularity in the gaming and entertainment sectors, its applications have since expanded to various fields, such as education, healthcare, and industrial training.

When VR technology first began to flourish, VR headsets needed to be connected to a computer to render frames of VR scenes, which are known as tethered headsets. With advancements in technology, such as powerful mobile processing, mature VR software platforms, and faster wireless connectivity, All-In-One VR (AIO VR) headsets have become the industry standard. These market-dominating VR devices include their own operating systems (OSs) within the headset. Recent examples include Meta Quest, PICO Neo, HTC Focus, and Apple Vision Pro. Compared to their wired counterparts, the AIO VR headset can perform all computing within itself, freeing users from the constraints of cables and allowing for portable, computer-independent usage scenarios.

Despite these advantages, AIO VR headsets come with some limitations. Smoothly rendering immersive VR environments requires substantial computing power and high-end hardware, which are difficult and costly to incorporate into the compact form factor of an AIO VR headset. Consequently, the accessibility and performance of VR experiences on standalone AIO devices may be limited due to this constraint. Another challenge for developers is the difference in operating systems compared to traditional, PC-connected VR applications, which are typically Windows-based. Migrating existing VR applications to AIO devices requires significant effort and resources to ensure compatibility and optimal performance, especially given the distinct OSs provided by different VR vendors. It becomes even more pronounced when dealing with embedded devices, as their firmware is highly dependent on the underlying OSs.

Recent advancements in multimedia streaming technology provide a solution to the hardware limitations of AIO VR headsets. Streaming technology enables users to experience high-quality VR content via the internet by shifting the heavy computational workload to remote servers. Instead of performing complex VR rendering locally, the user’s device only needs to parse input sensory signals and transmit them online. Accordingly, the VR content is generated in the cloud, then rendered and transmitted back to the user’s device to display in real time. By offloading these intensive computations to the cloud, users can access high-quality VR content on lower-end devices [1]. For consumers, this implies that purchasing high-end hardware is not necessary. For developers, VR streaming alleviates compatibility issues across various VR OS platforms, thus increasing development efficiency. This democratization of VR technology opens up new possibilities for both content creators and consumers, potentially leading to the widespread adoption of VR in everyday life. Additionally, rendering VR in the cloud enables users to interact with each other—cloud servers instantly render VR frames according to users’ poses and interactions, and synchronize to each user’s headset.

However, VR streaming also faces several challenges. One of the most pressing issues is the requirement for extremely high bandwidth and low latency to ensure responsive and high-quality VR experiences. Unlike traditional one-way 2D video streaming, VR frames must be transmitted according to the users’ pose in real time with minimal delay to maintain their immersion. Any noticeable lag can lead to motion sickness and disrupt the overall VR experience. In addition, VR streaming demands much higher network bandwidth to transmit high-resolution, 360-degree video that covers the user’s entire visual field. According to [2], data rates of more than 530 Mbps and a latency of less than 10 ms are required by high-quality VR, which are much more stringent than today’s general network applications.

On the VR headset side, current commercial VR streaming solutions are primarily built on the OpenXR standard, which registers as the active OpenXR runtime on the headset system to abstract hardware differences. It allows compatibility with existing applications using the standardized OpenXR API and eliminates the need to modify source codes. While offering convenience, it comes at the cost of reduced flexibility for developers. For instance, OpenXR lacks support for streaming custom sensory data, such as motion capture (MOCAP) or EEG data. This limitation poses a challenge for researchers aiming to analyze players’ behaviors or conditions via peripheral sensors in detail. Additionally, although OpenXR supports connections of multiple VR devices simultaneously, most video streaming systems do not inherently support action synchronization of multiple players, further constraining the potential for collaborative or multiuser experiences in VR.

**Figure 1:**
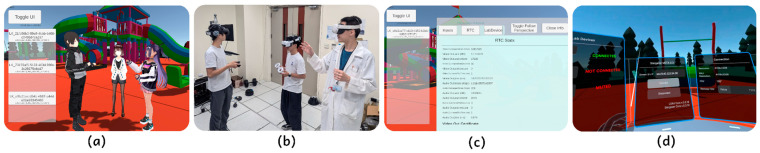
Loka is a reconfigurable VR streaming framework offering immersive scenes, local multiplayer support, and research data integration. The figure showcases the key components: (**a**,**b**) depict the real-time multiplayer experience from the host’s and real-world perspectives, (**c**) shows the administration panel for evaluating client performance, and (**d**) reveals the client’s interface.

In this paper, we propose Loka (https://github.com/ncu-wmlab/LOKA.Core, accessed on 22 January 2024), a VR streaming software toolkit developed using Unity Engine, a leading game engine widely used in VR application development. Loka integrates the benefits of AIO VR and streaming, allowing developers to avoid the complexities of cross-platform development and device-dependent SDKs. The toolkit provides a unified interface, handling the underlying specific organization of various VR OS platforms. We have tested Loka on various device models to ensure compatibility and performance. By eliminating the need for developers to “reinvent the wheel”, we aim to enhance VR application development efficiency, as well as extend its functions on integrating sensory data. Figure 1 illustrates the key components of Loka.

From the user’s perspective, Loka eliminates the need for high-end devices to experience high-quality graphics, which also conserves the storage space and energy consumption of devices as only a lightweight streaming app is required to gather and send input data, then processes the frames received from the server. Furthermore, our solution inherently supports multiplayer (multicasting) functionality, enabling developers to easily add users to the same virtual environment. For researchers, Loka can track a variety of data sources, including connection performance metrics and data from IoT devices linked to the VR headset, with the flexibility to extend support for additional data as needed.

## 2. Related Works

### 2.1. Network Technology

Network communication technologies have continuously evolved alongside advancements in transmission capabilities to meet the growing bandwidth demands and high-quality requirements of modern applications. The 5G mobile networks have enabled real-time high-quality video and audio streaming, offering peak throughput of up to 20 Gbps by allocating more spectrum. With a significant increase in capacity and efficiency compared to previous generations, 5G better supports a wide range of applications, including media streaming, cloud rendering, and interactions in VR and nano equipment, while maintaining high performance [3,4].

Looking ahead, cellular networks have already entered the Beyond-5G (B5G) era, meanwhile pursuing the next frontier: 6G. The International Telecommunication Union (ITU) released the IMT-2030 Framework through its vision, outlining the capabilities of 6G, which builds on the goals of IMT-2020 and introduces new innovations. IMT-2030 identifies six usage scenarios for 6G: three of them are extended from 5G, which are immersive communication, massive communication, hyper reliability and low latency communication (HRLLC), and the other three are ubiquitous connectivity, AI and communication, and integrated sensing and communication. VR/XR is one of the typical use cases of immersive communication. In immersive communication, mixed traffic of video, audio, and environment sensory data are transmitted in a highly reliable and low-latency time-synchronization manner. The feature can well support responsive and accurate interaction for real and virtual objects in VR. The other usage scenario envisaged in IMT-2030, integrated sensing and communication, facilitates new XR applications. It aims to offer spatial information about the movements and surroundings of unconnected objects as well as connected devices. Thus, we can imagine that VR/XR services are expected to be extensively applied in various vertical domains, such as education, medicine, and industry, to help people engage in activities and cooperate via networks [5,6].

### 2.2. Cloud Gaming

Cloud gaming has laid much groundwork for the technologies now applied in VR streaming. In cloud gaming, the computational tasks of rendering are shifted to remote servers, with video streams and user inputs exchanged over the Internet. For example, GamingAnywhere [7,8], proposed by Chen et al., is an open-source cloud gaming platform that has demonstrated the potential of cloud-based solutions in delivering high-quality gaming experiences to lower-end devices. It provides developers with a customizable framework for encoding, network transmission, and resource management, enabling flexibility in deployment across different devices which can be found in many research studies. Other commercial platforms, such as GeForce NOW [9], have advanced cloud gaming by leveraging large-scale infrastructure and sophisticated video compression techniques [10]. These platforms aim to provide low-latency, high-fidelity gaming experiences without requiring users to invest in high-end hardware [11]. While successful in gaming, the principles behind these platforms—offloading computation to the cloud and delivering results in real time—have heavily influenced the development of VR streaming technologies. However, the immersive nature of VR introduces greater complexity, such as volumetric content and low-latency interaction requirements, which necessitate new solutions.

### 2.3. VR Streaming

VR streaming expands on cloud gaming concepts but faces additional challenges due to the immersive and interactive nature of virtual reality. Existing VR streaming solutions, such as Quest Link and Virtual Desktop [12], allow users to stream high-quality VR content from a powerful PC to standalone VR headsets over local networks. Quest Link, which operates over a wired or wireless connection, and Virtual Desktop, a wireless solution, both rely on proprietary protocols optimized for low-latency video transmission and real-time interaction. These solutions offload computationally intensive tasks to external hardware while delivering responsive VR experiences to AIO headsets. An open-source alternative, ALVR (Air Light VR) [13], offers similar functionality but with greater flexibility for developers and researchers. Unlike some proprietary solutions, ALVR is open source, providing more customization options and enabling the community to contribute to its development. While it shares many of the benefits of proprietary platforms, ALVR’s open-source nature makes it an attractive option for developers looking for greater control over their VR streaming implementations.

However, the demands of VR streaming, such as the transmission of high-resolution, volumetric video, exacerbate the need for robust network infrastructures. Solutions like CloudXR from NVIDIA [14] have optimized VR streaming for these environments, offering low-latency experiences for both virtual and augmented reality applications. Nevertheless, current VR streaming platforms still face the challenges of multicast streaming and custom data collection. For instance, while OpenXR has enabled cross-platform compatibility for VR applications, it has limited support for transmitting specialized sensory data streams, such as EEG or motion data, which are essential for research-focused applications.

Loka is designed to fill critical gaps in existing VR streaming solutions by providing self-hosting, full VR support, and custom data integration for immersive applications development. Existing solutions, such as GamingAnywhere, Google Immersive Stream for XR, and NVIDIA CloudXR, provide valuable functionality but lack certain critical features necessary for VR-specific research workflows, such as multicasting capabilities, and flexibility for custom sensory data. As summarized in Table 1, Loka’s modular framework uniquely addresses these gaps.

### 2.4. IoT Sensor Integration

In research, VR experiments are often conducted by integrating built-in or wearable embedded sensors, with the aim to enhance immersion via obtaining perceptive feedback in the physical world [15]. For instance, Rosu et al. integrate BLE beacon data to synchronize the state of a real-world device with its digital twin [16]. Similarly, Mancuso et al. utilized multiple sensor data streams collected during a VR session to perform psychometric assessments. Their research leveraged skin conductance (SC), surface electromyography (sEMG), and photoplethysmography (PPG) biosensors, in addition to built-in sensors for eye tracking and head movement, providing a depth of data for analysis [17].

Various commercial products can collect different types of data to enhance the VR experience. One of the most interesting fields is motion capture (MOCAP), which, in particular, has become a focal point for data collection and real-time interaction for both entertainment and research purposes. To this end, Sony introduced Mocopi, an array of compact sensors which can capture users’ arm, torso, and leg movements in real time [18]. HTC has also introduced a range of trackers that provide diverse data, including eye tracking, facial tracking, and motion capture information [19].

To achieve the most immersive VR or Metaverse experience, real-time biofeedback integrated into the simulated environment is highly preferable. However, a significant challenge is that, in most research contexts, sensor data are often collected separately from the game module itself. This can lead to synchronization issues and a lack of immediate feedback. Additionally, the integration of biofeedback systems faces challenges related to privacy concerns and compatibility across different VR devices [20].

## 3. Objectives and Features

In this paper, we proposed Loka, a novel VR streaming toolkit designed to overcome the limitations of existing solutions by integrating the benefits of AIO VR and cloud VR streaming. Our work offers several key contributions to both developers and researchers, enabling them to efficiently create and deploy interactive, cross-platform VR applications with ease. The main features and contributions of Loka include the following:

- Cross-platform compatibility without custom SDKs: Loka simplifies cross-platform development by abstracting away device-specific SDKs, allowing developers to build VR applications without worrying about platform-dependent differences. By providing a unified interface, it reduces the time spent on reconfiguring applications for different hardware environments.
- Seamless integration of custom data streams: Unlike current VR streaming platforms, Loka supports the integration of custom data streams, such as motion data, EEG data, and physiological signals from IoT devices. This makes it ideal for research applications, enabling researchers to gather a wide range of data for behavioral analysis, neuroscience experiments, or physiological monitoring within immersive environments.
- Multiplayer and multicasting support: Loka natively supports multicasting functionality, allowing multiple users to interact within the same virtual environment on the same machine, which many existing platforms could not achieve. This facilitates collaborative experiences or large-scale Metaverse experiments where multiple users are involved in real time.

### 3.1. Applications of Loka

Loka’s extensibility allows it to support emerging applications, such as immersive virtual workspaces for remote teams. Leveraging its self-hosted and customizable nature, researchers and developers can create solutions tailored to specific use cases. Two key types of applications are illustrated below:

- Content personalization: Loka’s modular architecture and support for integrating sensory data make it well-suited for developing personalized VR experiences. It enables the creation of personalized VR training programs where the difficulty or content adapts in real time based on the user’s physiological responses, such as eye movement or stress levels. Delivering immersive entertainment experiences tailored to individual preferences, such as adjusting the VR environment based on user behavior or interests, is another interesting application.
- Industrial solutions: Loka can facilitate remote VR collaborative environments for design and prototyping, allowing multiple users to interact with 3D models or virtual machinery in real time. By integrating IoT sensor data, Loka can be used for real-time monitoring and control of industrial processes in VR, improving efficiency and reducing downtime. Loka’s support for custom sensory data makes it ideal for applications like remote diagnostics, where doctors can use tactile and visual feedback from IoT-enabled devices to interact with patients remotely.

### 3.2. Cost-Effectiveness of Loka

Loka’s self-hosted and modular architecture offers significant cost advantages compared to many commercial VR streaming solutions. Unlike proprietary systems such as NVIDIA CloudXR, which require specific hardware (e.g., NVIDIA GPUs) and licensing costs, Loka is designed to be hardware-agnostic and open-source, making it accessible to researchers and industries with diverse setups. We compare Loka with similar solutions described in Table 1 in terms of financial complexity across the following key aspects:

- Hardware requirements: Loka does not depend on specialized hardware, allowing organizations to leverage existing infrastructure, which reduces initial setup costs. Competing solutions like NVIDIA CloudXR often require high-performance GPUs.
- Licensing costs: As an open-source framework, Loka eliminates the licensing fees typically associated with commercial platforms such as Unreal Engine-based solutions or NVIDIA CloudXR, offering a more economical alternative. In addition, it allows organizations to independently maintain and update the system, which avoids vendor lock-in and eliminates recurring subscription fees.
- Customization and scalability: The modular design of Loka allows for easy customization, enabling industries to adapt the framework to their specific needs without incurring significant development costs. Moreover, its self-hosting feature minimizes reliance on external cloud services, reducing ongoing operational costs.

## 4. System Architecture

**Figure 2:**
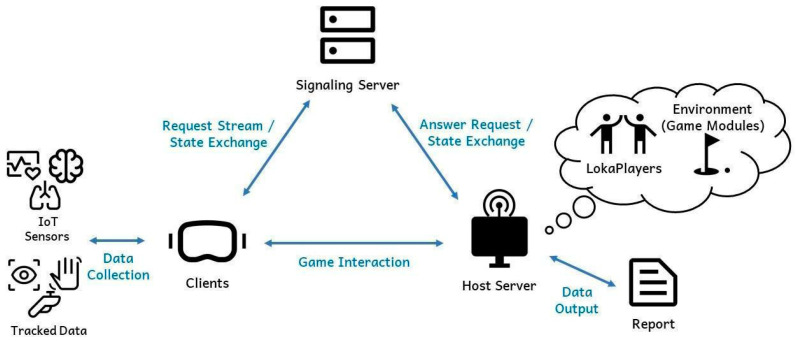
Loka’s deployment architecture.

Loka’s architecture is designed to provide a robust VR streaming solution, allowing for real-time interaction, data collection, and multiplayer functionality. As illustrated in Figure 2, the system comprises several key components, including clients, which are VR devices connected with peripheral sensors, a signaling server, and a host server. The architecture facilitates the seamless data flow between these components by leveraging Web Real-Time Communication (WebRTC) for low-latency communication. This setup enables real-time VR streaming, custom data integration, and multiplayer support, all in a highly flexible environment.

### 4.1. Client: VR Devices and Data Collection

The client typically denotes the VR devices worn by users. These devices are responsible for receiving streamed frames from the host server and transmitting real-time user inputs to the server. In addition to tracking basic interaction data (e.g., head movements and controller inputs), the system supports the collection of more advanced data streams in real time, such as tracked data and IoT sensor data. The former denotes motion tracking data supported by devices; the latter collects custom data streams from peripheral embedded IoT devices, such as physiological sensors.

These data are continuously sent to the host for processing and rendering, which could also be retrieved by the virtual scene in real time, enabling a responsive and interactive VR experience while also supporting research applications that require detailed data tracking.

**Figure 3:**
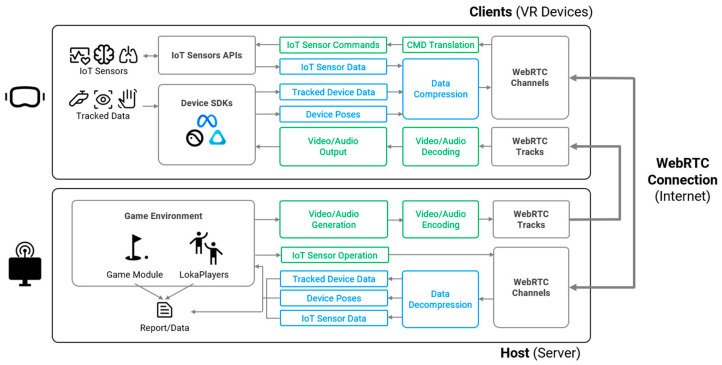
Streaming components of Loka: The client (VR devices) collects real-time IoT sensor data, tracked device data, and poses via IoT sensor APIs and device SDKs. The data are compressed, decoded, and transmitted to the host over WebRTC channels. The host decompresses and processes the data, updates the game environment, generates video/audio content, encodes, and transmits the data back to the client via WebRTC tracks for real-time rendering. Reports and data logs are also generated for further analysis. IoT sensor commands are issued and translated on the client side, helping to decouple the device model for improved compatibility.

As illustrated in Figure 3, Loka processes data on the client side by categorizing them into a standardized format before transmitting the formalized data to the host. When integrating new sensors or VR devices into Loka, developers can utilize the two groups of modules located in the top-left corner—sensor API and device SDK—to connect to the relevant functions within Loka. Depending on the specific sensors and devices, it may be necessary to extend these modules to transform the data into the required format. This modular design minimizes effort and eliminates the need for extensive modifications to the overall architecture, ensuring seamless and efficient integration.

### 4.2. Host: Rendering Server

The host server is the backbone of Loka’s architecture, managing the environment (game modules) and handling computationally intensive tasks, such as rendering. The server is capable of handling multiple clients simultaneously, each represented as LokaPlayers, interacting within the same virtual environment. It processes incoming data from the clients and generates the corresponding frames to be sent back to the devices. The host server is also responsible for the following:

- Environment management: run the virtual environment, ensuring that all users experience a synchronized, immersive experience.
- Custom data handling: process specialized data streams, such as physiological signals and IoT data, for real-time integration into the VR experience.
- Data and log creation: collect users’ input and generate reports and systematic logs for further analysis, such as player behavior or research-related metrics.

### 4.3. Signaling Server: Signal Communication

Loka employs a signaling server to establish and maintain communication between the clients and the host. This server is integral to setting up peer-to-peer (P2P) connections via WebRTC, which handle real-time signal exchange. The signaling server is responsible for the following:

- Host–server matching: The signaling server matches available host servers for clients based on their connection requests. When a client device initiates a connection, the signaling server selects an appropriate host server to handle the session.
- Signal exchange: The signaling server is responsible for the exchange of signaling information, which is required to establish a connection between the client and host. This includes exchanging available connection methods and the state of the client–host connection.

The detailed steps involved in the connection process will be further discussed in the next section.

## 5. Technical Implementation

Loka (the source code is publicly accessible from GitHub: https://github.com/ncu-wmlab/LOKA.Core, accessed on 22 January 2024) is built atop Unity Render Streaming (URS). This experimental yet powerful framework leverages real-time rendering in Unity and facilitates remote content delivery, and its source code is publicly reachable on GitHub [21]. At the core of URS’s data transmission is WebRTC, which enables low-latency, real-time data exchange between the client and server, ensuring smooth interactions and responsive VR experiences.

To support cross-platform VR functionality, Loka integrates the Unity XR Interaction Toolkit along with the OpenXR Plugin, ensuring that the system can be deployed across various VR devices without needing significant modifications to the application code. This approach abstracts device-specific dependencies and allows developers to seamlessly integrate different VR hardware into their applications. On the client side, Loka interfaces with device-specific SDKs to ensure full compatibility with the unique features of each VR headset, such as Quest, PICO, and other popular AIO VR devices. The framework’s modular design enables Loka to be efficiently extended to accommodate new devices and hardware environments. Additionally, automated compatibility testing pipelines can be incorporated to ensure seamless cross-platform performance as new devices and operating systems emerge.

### 5.1. Establishing Connection

**Figure 4:**
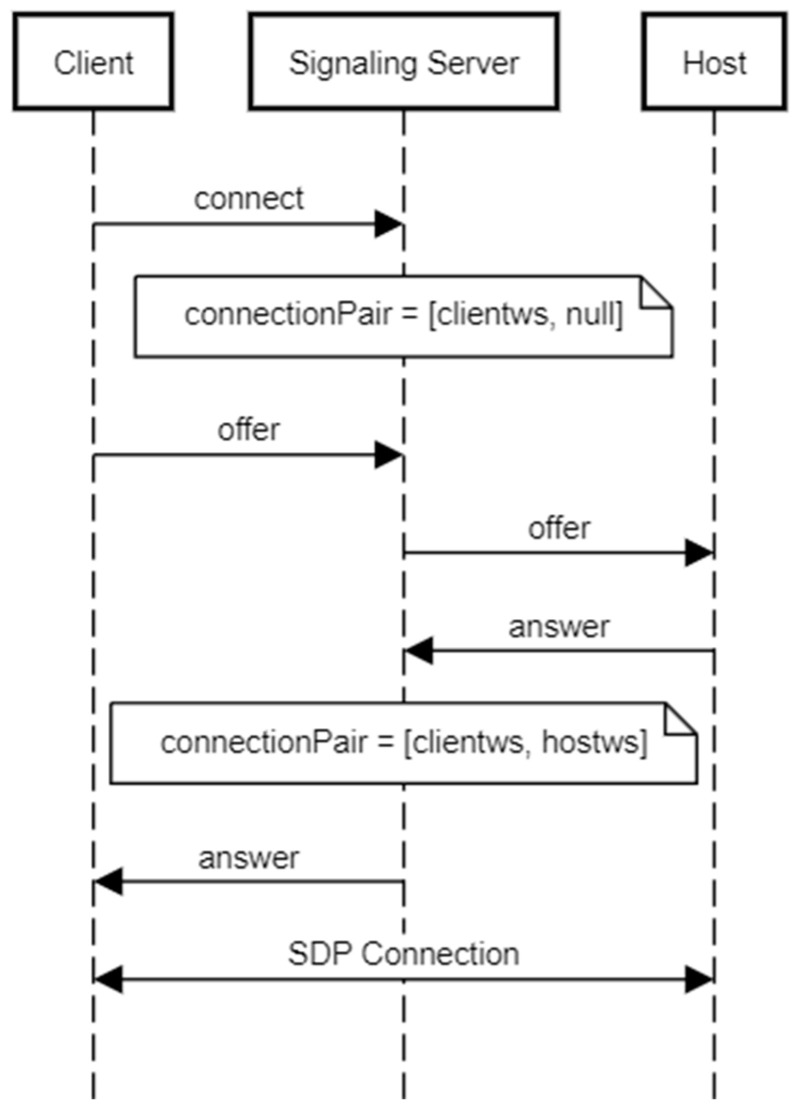
Sequence diagram for establishing a connection between the VR client and the host.

The connection between the client and the host is established through the signaling server, following the standard WebRTC connection setup process. We extended the example web app of URS written using Node.js. It achieves its role as a WebSocket server, which is responsible for establishing and maintaining WebRTC connections across multiple devices. As shown in Figure 4, the client first initiates the connection by sending a connect request to the signaling server. Upon receiving the request, the signaling server creates a connectionPair, initially linking the client’s WebSocket connection. Next, the client sends an offer to the signaling server, which is forwarded to the host. The host then responds with an answer, which the signaling server relays back to the client. Once the answer is received by the client, the signaling server updates the connectionPair to effectively pair the two. After both parties exchange their offer and answer messages, the Session Description Protocol (SDP) connection is established, allowing real-time data exchange to begin between the client and the host.

### 5.2. Host View Adaptation and Rendering

Once the connection is established, the host dynamically adjusts the field of view (FOV) to match the client’s FOV settings. This ensures that the visual experience on the client’s VR device aligns with the intended perspective. The FOV can still be adjusted during runtime, allowing for seamless adaptation to user preferences or device constraints.

The host captures the in-game view from the perspective of the LokaPlayer—the virtual representation of the user in the virtual scene. This view is then rendered into an image with an aspect ratio of 2:1. The rendered image is then transmitted to the client and displayed in front of the user. If the client’s display size does not match this ratio, the system automatically adjusts and fits the image to ensure a consistent and clear visual presentation without distortion. For instance, Meta Quest 3 has a resolution of 4128 × 2208; therefore, the rendered frame will be displayed in 4416 × 2208.

### 5.3. Client Input Handling

Loka’s input system is built on Unity’s new Input System, which offers a flexible and modular way to handle user input. This system separates input into devices and controls. Each device (e.g., controller) consists of multiple controls (e.g., button presses, joystick movements, device position). The input is abstracted into input actions and input action maps, where actions listen for changes in the corresponding controls to determine whether an action is triggered or to retrieve specific input values. On top of that are input action assets, which serve as the container of action maps and could be serialized as files.

**Figure 5:**
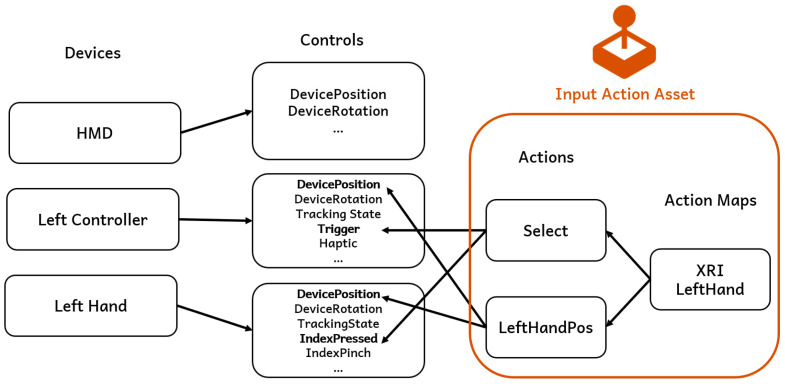
Integrated input system architecture.

As illustrated in Figure 5, an action map can contain multiple actions. For example, the action Position in action map XRI LeftHand is configured to detect the position data from either the Left Controller or Left Hand (captured by hand tracking) by looking for the appropriate controls within those devices. We also provide ControllerPos and HandPos to let developers retrieve the dedicated data. This system allows for a flexible mapping of device inputs to in-game actions, which is particularly useful in VR environments with multiple input devices.

Loka extends this system by synchronizing the input system from the client to the host machine via a WebRTC data channel, ensuring that the host can receive all relevant input data from connected VR devices. This approach allows the host to mirror the actions and input values of each player, ensuring that the virtual environment behaves consistently based on the real-time actions of each user.

WebRTC data channels support buffering of outbound data, enabling real-time monitoring of the buffer state. Notifications can be triggered when the buffer begins to run low, allowing the system to ensure a steady stream of data without memory overuse or channel congestion. This mechanism is critical in a dynamic multiplayer environment, as it minimizes input delays, prevents data loss, and ensures synchronization accuracy for players.

Loka uses a “first-touch” mechanism to handle simultaneous input on the same virtual object, relying on the queued sequence of the WebRTC data buffer to determine input priority. While network latency can influence input timing, for research located on a local network, as in our experimental setup described in Section 6, the difference in input timing can be negligible.

**Figure 6:**
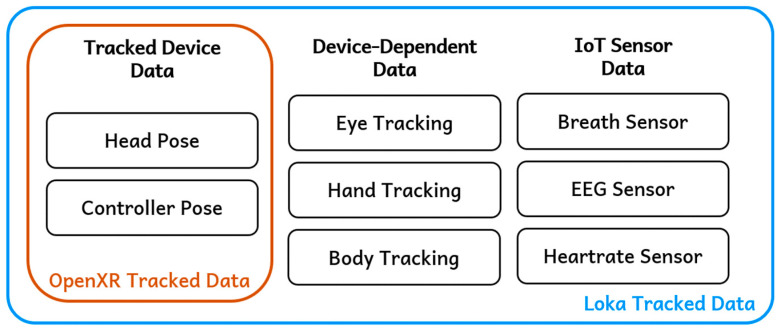
Different types of tracked data.

In conventional VR streaming solutions that rely on OpenXR runtime, the supported input signals are typically limited to fundamental data such as the poses of heads or controllers (Figure 6). These signals are essential for the basic VR interaction supported by most devices. However, this restricts the ability to integrate device-specific or advanced input features, such as eye tracking or EEG sensors. Loka addresses this limitation by extending input systems to support a wider range of data types. For instance, device-dependent data like eye tracking and hand tracking are fully integrated into the framework. In addition, since the device-dependent data are not standardized, the same type of data collected from each device may use varying nomenclatures.

To address this problem, Loka interprets the data on the client side, categorizes them into a standardized format, and then transmits the formalized data to the host. Beyond device-specific capabilities, Loka supports the real-time integration of custom IoT sensor data. For devices with unique input formats, Loka employs adapters to preprocess and map raw signals to the framework’s standardized format, maintaining compatibility across various VR headsets and IoT sensors. Also, since some sensors require a startup signal to work, Loka reserves a direct channel to allow server–sensor communication (as Figure 3 depicts). These capabilities provide researchers and developers with a versatile and customizable environment, facilitating advanced applications such as biofeedback systems and personalized user interactions.

Loka standardizes input signals across devices by translating device-specific formats into a unified internal representation. This is achieved through Unity’s Input System and XR Interaction Toolkit, which manage inputs dynamically and ensure consistent interaction experiences. For devices with unique input formats, Loka employs adapters to preprocess and map raw signals to the framework’s standardized format, maintaining compatibility across various VR headsets and IoT sensors.

### 5.4. Multicasting Capability

Unlike typical streaming or PCVR solutions, Loka natively supports multiuser functionality within a single host server, allowing multiple users to interact in the same virtual scene simultaneously. This capability creates opportunities for VR research in areas such as social interaction, a key component of the Metaverse. This functionality is achieved by decoupling the OpenXR runtime from our solution. Since the OpenXR runtime does not support multiple devices connected to the same computer simultaneously, we use it solely to translate input signals. The actual input data are transmitted via the WebRTC channel to the host, as described earlier.

**Figure 7:**
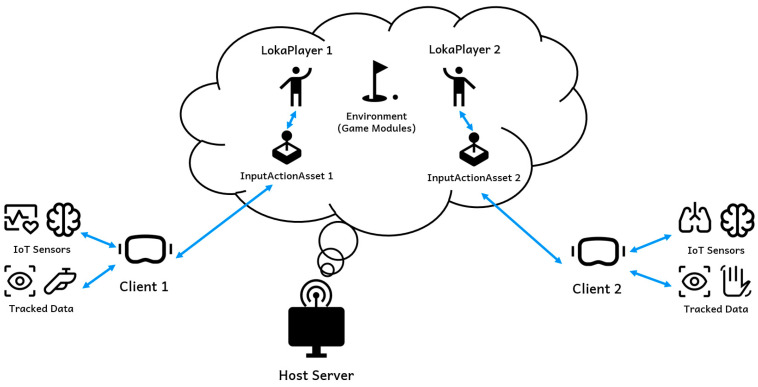
Multiuser architecture in Loka. The host server supports multiple clients, each operating on different platforms or equipped with unique IoT sensors and controllers.

Since Loka supports multiple users in the same scene simultaneously, we developed a custom action map in the host, which stores various types of VR inputs, including controller poses and eye-tracking data. Each time a player connects to the system, a cloned version of this action map is assigned to the player’s corresponding LokaPlayer instance. By binding the player’s inputs to their respective action map, Loka ensures that each player’s inputs are correctly tracked and processed in real time. Whenever a player’s input is updated, the system reads the bounded action map to retrieve the correct values, providing precise input handling for multiplayer VR environments. This architecture is illustrated in Figure 7, where the Input Action Asset defines actions and action maps for each player. The LokaPlayer on the host is assigned a cloned action map, which ensures that input data (e.g., device position, rotation, button presses) are accurately reflected in the host environment.

## 6. Results

In this section, we evaluated the performance and effectiveness of Loka. We conducted a series of tests focused on key areas such as networking load or responsiveness under different scenarios.

### 6.1. Testbed Setup

Our experiments were conducted on a controlled testbed within our lab. To simplify the network topology, we ran the host and signaling server on the same machine, as the signaling server only functions as a state exchanger. The machine used was a Windows 11 desktop, equipped with an Intel i7-13700K processor and an NVIDIA RTX 4060 Ti graphics card. The streamer program is built on Unity 2020.3.33f1.

To facilitate performance analysis, we implemented a logging system in the Loka framework, which records connection metrics during runtime. These metrics include WebRTC-specific performance indicators such as latency, packet loss, and bitrate. The metrics can be monitored on the host machine in real time and are also saved to the file system for later analysis. The recorded data will be used to evaluate system performance under different conditions. For instance, the logging system can be used to study Loka’s performance under high-frequency data input scenarios by monitoring real-time CPU usage and tracking latency variations in relation to the volume and frequency of input data.

On the client side, we used the PICO Neo 3 Pro Eye (PICO Interactive, Beijing, China) throughout the initial experiment, which natively supports eye tracking. Additionally, several IoT sensors were integrated into the setup, including a breath sensor and an EEG sensor. To evaluate cross-platform compatibility, we replicated the experiment on other VR devices, including Meta Quest Pro (Meta Platforms, Menlo Park, CA, USA) and Meta Quest 3 (Meta Platforms, Menlo Park, CA, USA), as well as on PC platforms. These additional experiments aimed to assess Loka’s performance across various platforms and devices, which are detailed in Section 6.3.

The breath sensor is an Arduino board (Arduino, Turin, Italy) equipped with a barometer, mounted on a belt. During the experiment, the user wore the belt around the abdomen. When the user inhales, the abdomen will expand, causing the barometer reading to increase; conversely, when the user exhales, the reading decreases. The sensor readings are transmitted to the Arduino board and then to the headset via Bluetooth (BT). In our setup, we use an Arduino Uno or Arduino Leonardo for the breath sensor. Since these boards do not have built-in BT functionality, we include an HC-06 module (PiePie, Taipei, Taiwan) for BT data transmission. The HC-06 module is connected to the Arduino via its serial interface (RX-TX); once the barometric sensor data are read by the Arduino, they are wirelessly relayed through the HC-06, which sends the data to the headset.

**Figure 8:**
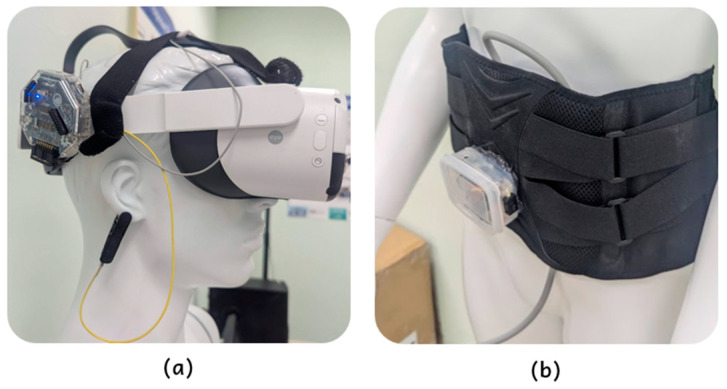
Integrated IoT sensors. (**a**) Ganglion board. (**b**) Breath sensor.

For the EEG sensor, we utilized Ganglion, a commercial Arduino-based bio-sensing device compatible with OpenBCI (Figure 8). Ganglion is capable of monitoring EEG, EMG, or ECG signals, with data sampled at 200 Hz on each of the four channels. The sensor was integrated with the headset to collect real-time brainwave data during the experiment. Ganglion uses Bluetooth Low Energy (BLE) to transmit data in a specialized format. To enable seamless integration with the VR environment, we developed an embedded library in Android Studio and incorporated it into Unity (the implementation (Android Studio): https://github.com/ncu-wmlab/LabFrameAndroidPlugins/tree/master/ganglion_plugin/src/main/java/com/xrlab/ganglion_plugin, accessed on 22 January 2024) (the interface code in Unity: https://github.com/ncu-wmlab/LabFrame_Ganglion, accessed on 22 January 2024), allowing real-time data streaming at 200 Hz.

### 6.2. Bandwidth Loads

Loka is built atop WebRTC, whose congestion control capability can dynamically adjust the target video bitrate based on the estimated network throughput and condition. It begins with an initial setting and efficiently adjusts in real time as the network fluctuates. WebRTC receivers can send receiver estimated maximum bitrate (REMB) messages to the sender as soon as they detect any congestion and then keep sending the messages per second even if no congestion is happening. Then, the sender decides if the transmission bitrate can be raised or should be immediately lowered. REMB messages are usually generated by the receivers every 250 to 500 ms [22]. Consequently, dynamic forwarding operates on a fine-grained timescale, effectively accommodating short-term bandwidth variations among receivers and ensuring seamless playback continuity. The bandwidth adaptation feature is ideal for remote cooperation scenarios. Gunkel et al. [23] presented a WebRTC-based system for photorealistic social VR communication and evaluated the performance of the system for handling multiple user streams.

Another key feature is the adaptive bitrate capability of the AV1 encoder, which is particularly effective in reducing redundant data in low-motion or static frame scenarios, significantly optimizing bandwidth usage. Compared to traditional codecs, AV1 delivers approximately 30–50% better compression efficiency than H.264 and 20–30% better than H.265 (HEVC) while maintaining the same level of visual quality. Uhrina et al. [24] analyzed the compression performance of several modern codecs, revealing that their efficiency varied with resolution. Notably, newer codecs like AV1 demonstrated greater efficiency at higher resolutions. These findings highlight the potential advantages of using the AV1 codec for VR streaming, which typically demands high-resolution content. This advantage enables Loka to optimize bandwidth consumption while preserving high visual fidelity. It can adapt to lower bitrate for low-complexity scenes, such as minimal movement or changes, to reduce bandwidth usage, but also keep the visual quality. Loka’s integration of WebRTC and AV1 ensures adaptive data transmission in dynamic environments. By monitoring network conditions and adjusting bitrate dynamically, the system reduces latency and prevents bandwidth overuse while maintaining high visual quality.

**Figure 9:**
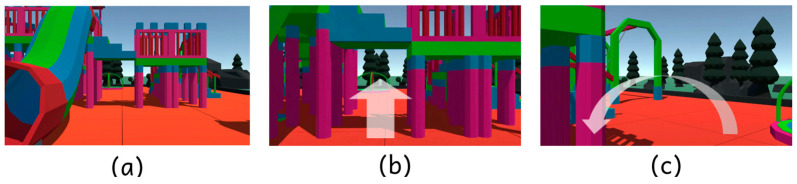
Visual representation of the three experimental phases. (**a**) Phase 1: the user remains stationary. (**b**) Phase 2: the user moves forward, navigating through the playground. (**c**) Phase 3: the user freely explores the scene, including rotational movements.

To evaluate this adaptive characteristic, we designed our experiment in three phases (Figure 9). Each phase was designed to assess network performance under different levels of user movement and scene complexity:

- Phase 1: The user was instructed to remain still, looking straight ahead with minimal head or body movement. This phase simulates a low-motion scenario, allowing us to measure our ability to maintain high-quality streaming when only minor frame updates are necessary.
- Phase 2: The user was asked to move forward within the virtual environment, specifically walking through a playground slide. This phase represents moderate user movement, introducing more complex frame changes as the user interacts with objects in the scene.
- Phase 3: In this phase, the user was allowed to freely walk and turn around in the scene. This phase introduced both rapid movement and changing perspectives as the user explored the virtual environment. With significant changes to both the objects and the user’s perspective, this phase poses higher bitrate demands and more frequent frame updates.

**Figure 10:**
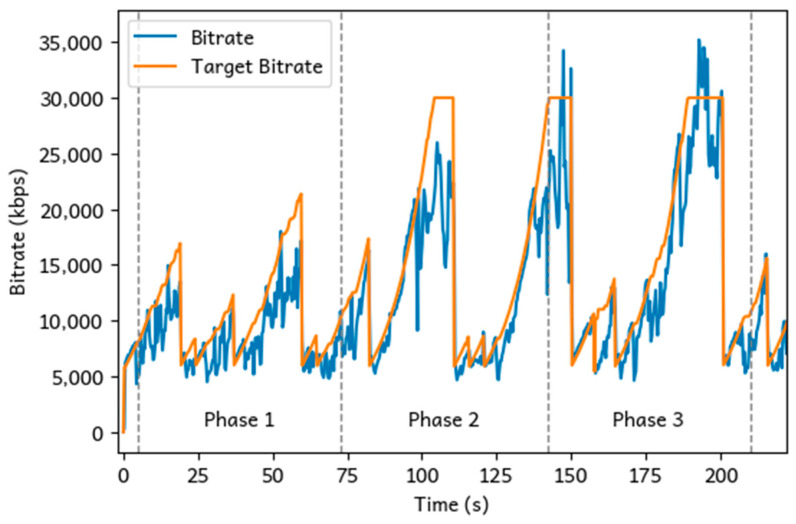
Bitrate performance and the target bitrate WebRTC evaluated across three different use cases.

Figure 10 demonstrates how WebRTC responded to different scenarios across the three phases. During the low-motion scenario, where the user remained mostly still, the bitrate remained relatively low and stable at an average of 8353 kbps. This is expected in scenarios with minimal movement, as WebRTC saves up bandwidth by reducing the number of frame updates. As the user began to move in the virtual environment, the bitrate increased significantly. The average bitrate during this phase rose to 12,536 kbps, reflecting the more complex frame updates required to handle moderate user movement. WebRTC adapted by dynamically increasing the target bitrate to ensure high-quality streaming in response to the increase in scene complexity. Finally, in phase 3, the user was allowed to freely walk and turn around, introducing rapid movement and more frequent changes from the user’s perspective. The system reported a further increase in bitrate, averaging 14,675.5 kbps. The higher bitrate in this phase reflects the increased demand for frequent frame updates to accommodate the dynamic state of the scene. Despite the increased motion, the system managed to maintain a relatively stable frame rate and avoid significant delays.

### 6.3. Multi-Platform Performance

Loka’s cross-platform compatibility is enabled by its modular design and reliance on Unity’s XR Interaction Toolkit and OpenXR Plugin. This approach abstracts device-specific dependencies and ensures seamless operation across different hardware. Rigorous cross-platform testing, including evaluations on various VR devices and operating systems, ensures consistent performance. The framework’s dynamic configuration capability further enhances adaptability, enabling Loka to accommodate new devices and platforms efficiently.

To evaluate Loka’s performance across various platforms and devices, we replicated the experiment mentioned in the previous section on a range of VR devices, including PICO Neo 3 Pro Eye, Meta Quest Pro, and Meta Quest 3 (Table 2). In addition to VR devices, we conducted the experiment on PC platforms, where keyboard inputs were used to simulate the control of movement and viewport, to mimic the VR experience. The PC tests were performed on both Windows and MacOS systems to assess Loka’s compatibility and performance across desktop environments. By covering a diverse range of platforms, both VR and non-VR, we ensured that Loka’s performance was evaluated under various hardware and software configurations, providing a comprehensive understanding of its streaming capabilities across different devices and operating systems.

**Figure 11:**
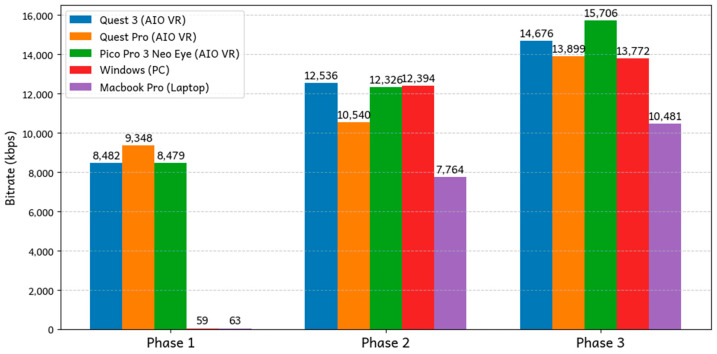
Average bitrate across different devices.

The result is depicted in Figure 11, where we can observe a clear trend in bandwidth consumption across the three phases. Phase 3, which involved the most user movement and scene complexity, consistently consumed the most bandwidth, followed by Phase 2 with moderate movement, and finally Phase 1, where users were mostly stationary. This pattern aligns with the results from the previous section, reinforcing that higher user motion and scene complexity demand greater bandwidth for real-time streaming in VR environments.

A notable observation is the lower bitrate performance on PC platforms (Windows and MacOS) during Phase 1 compared to the VR devices. This discrepancy can be attributed to the fact that, on PCs, users could remain completely stationary, while VR users, though instructed to remain still, could still exhibit subtle, involuntary movements such as head tilts or minor body shifts. These slight movements were enough to increase the bitrate required for VR streaming, as even small changes in position result in new frame data being sent. Additionally, the use of the AV1 codec could play a significant role in this observation. AV1 is known for its exceptional efficiency in handling low-motion or static frames, significantly minimizing the bitrate when there are no major changes in the scene [25]. As a result, on PC platforms where users could remain truly stationary, the bitrate remained consistently low during Phase 1. This highlights AV1’s ability to optimize bandwidth usage in low-motion scenarios, particularly when streaming static frames.

Overall, the results demonstrate Loka’s ability to adapt to different platforms and scenarios, efficiently managing bandwidth across varying levels of motion and scene complexity. The system’s performance across multiple platforms shows that Loka can effectively scale its streaming capabilities to meet the demands of different devices and demands.

### 6.4. Multicasting Performance

To evaluate Loka’s multicasting capability, we conducted an experiment by connecting multiple clients to a host simultaneously. The test began by connecting a single device to the host, followed by the addition of a new device every 60 s. Throughout the process, we measured the bitrate and framerate performance to evaluate the system’s behavior under increasing load.

**Figure 12:**
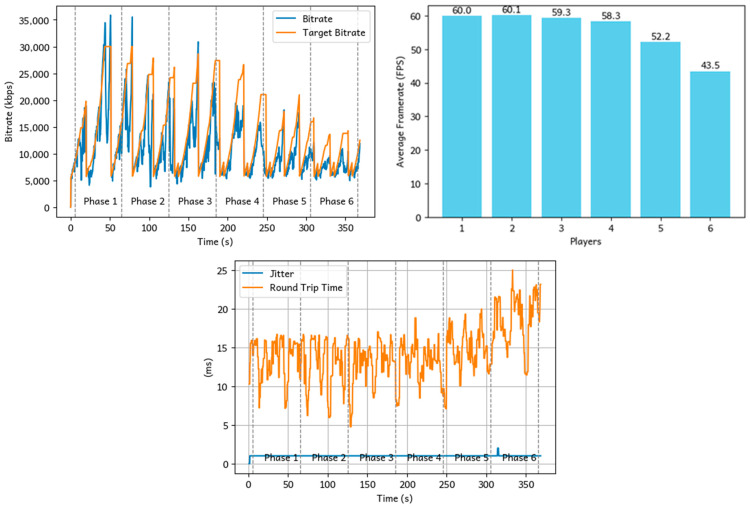
Average bitrate, frame rate, and latency performance with multiple devices connected to the host.

The results of the multicasting performance experiment are shown in Figure 12. We can observe that the frame rate remains stable (i.e., close to 60 FPS) for up to three players interacting in the same virtual environment at the same time, demonstrating that the system is able to perform smoothly without impacting users’ experience. However, as the number of players increases, the frame rate gradually declines, dropping to between 40 and 50 FPS with six players. Additionally, the maximum round-trip time for packets increases from 15 ms to 25 ms. This performance degradation is primarily attributed to CPU load, as the system requires significantly more computational resources to process and synchronize data as more players join.

This performance drop is primarily attributed to CPU resource limitations on the host server. As more players join, the system requires significantly more computational resources to process and synchronize data streams, leading to increased CPU load. Consequently, the system becomes CPU-bound, and this serves as the major bottleneck in the current experimental setup.

The results indicate that, on our experimental host hardware, Loka performs efficiently in multiplayer settings with up to three concurrent users, maintaining consistent frame rates, lower latency and delivering a seamless experience. However, beyond this threshold, performance degradation occurs. The WebRTC testing conducted in [26] also highlights the impact of resource limitations on performance. The service was hosted on a medium-sized cloud instance with 2 vCPUs and 4 GB of RAM. The study found that the system could support up to approximately 175 clients while maintaining acceptable latency. However, when the number of connected clients exceeded this threshold, the latency for all connected clients increased dramatically.

To address this limitation, a straightforward solution is to scale the system by increasing the CPU resources on the host server to support additional multicasting sessions. Furthermore, future optimizations will prioritize the development of efficient resource allocation strategies to enhance CPU utilization and improve overall system scalability.

## 7. Conclusions and Future Works

In this paper, we introduced Loka, a VR streaming toolkit designed to support the growing demands of the Metaverse by integrating the flexibility of AIO VR with the power of cloud-based rendering and streaming. Loka enables high-quality VR experiences on low-end hardware by offloading rendering tasks to remote servers, overcoming hardware limitations while supporting a variety of platforms. We assessed Loka’s ability to adapt to varying network conditions and user motion, ensuring stable and immersive experiences. Additionally, Loka’s multicasting functionality allows multiple users to engage in real-time interactions, a crucial feature for expanding social and collaborative experiences in the Metaverse, while integrating IoT sensor data, further enriching VR applications for both developers and researchers.

For future work, we plan to integrate Loka into our existing game modules, expanding its cross-platform and multiplayer capabilities. This integration will allow for seamless interaction between players on different devices, enhancing the versatility of the system. In addition, we plan to test Loka in various real-world scenarios to demonstrate its applicability and performance in practical settings. For example, in a remote education scenario, Loka will be used to create immersive VR classrooms where multiple students can interact in real time. These tests will evaluate key performance metrics such as synchronization accuracy, latency, and the effectiveness of personalized content delivery in enhancing learning outcomes. From a technical standpoint, we aim to study approaches to optimize bandwidth consumption and reduce latency by exploring advanced techniques such as foveated video streaming, which achieves throughput reduction by prioritizing rendering quality in areas where the user is looking [27,28]. The user’s viewpoint prediction can be also utilized to cache video data proactively and partially offload computing tasks to the edge server, to meet the demanding E2E latency [29]. Additionally, we plan to implement quality of service (QoS) prediction algorithms to instantly predict the variance of the demanded quality metrics and allocate resources accordingly, to ensure a smooth streaming experience even under fluctuating network conditions [22]. We plan to design adaptive algorithms for input prioritization, such as dynamic down-sampling of low-priority data streams and resource allocation strategies for high-priority inputs, to ensure that high-frequency input data do not degrade system performance and user experiences in multiplayer environments.

## Figures and Tables

**Table 1:** Comparison of open source and commercial solutions on Loka’s unique features.

	Supports VR	Self-Host	Eyetrack Data	Custom Data Stream	Multicasting
GamingAnywhere	No	Yes	No	Yes	No
Google Immersive Stream for XR	No	No	No	No	No
OpenXR-based tools (e.g., ALVR)	Yes	Yes	No	No	No
NVIDIA CloudXR	Yes	Yes	No ^1^	No	No
Loka (our work)	Yes	Yes	Yes	Yes	Yes

^1^ Planned support in future versions.

**Table 2:** The VR device used within the experiments.

	PICO Neo3 Pro Eyes	Meta Quest Pro	Meta Quest 3
Preview	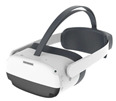	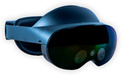	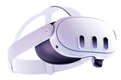
Resolution	3664 × 1920	3600 × 1920	4128 × 2208
FOV	95°	98°	100°
Connectivity	Wi-Fi 6	Wi-Fi 6E	Wi-Fi 6E
Built-in Sensor Data	Eye tracking	Eye tracking Hand tracking	Hand tracking
Integrated Embedded Sensors	EEG sensor (Ganglion) Breath sensor	EEG sensor (Ganglion) Breath sensor	Breath sensor

## Data Availability

The data presented in this study are openly available in Source Code at https://github.com/ncu-wmlab/LOKA.Core (accessed on 22 January 2024).

## References

[1] Wang H., Martinez-Velazquez R., Dong H., El Saddik A. (2024). Experimental Studies of Metaverse Streaming. IEEE Consum. Electron. Mag..

[2] Kizilkaya B., Zhao G., Sambo Y.A., Li L., Imran M.A. (2021). 5G-enabled education 4.0: Enabling technologies, challenges, and solutions. IEEE Access.

[3] Latha D.H., Reddy D.R.K., Sudha K., Mubeen A., Savita T.S. (2014). A Study on 5th Generation Mobile Technology-Future Network Service. Int. J. Comput. Sci. Inf. Technol..

[4] Yu H., Lee H., Jeon H. (2017). What is 5G? Emerging 5G mobile services and network requirements. Sustainability.

[5] (2024). Framework and Overall Objectives of the Future Development of IMT for 2030 and Beyond. https://www.itu.int/dms_pub/itu-d/oth/07/31/D07310000090015PDFE.pdf.

[6] Saad W., Bennis M., Chen M. (2019). A vision of 6G wireless systems: Applications, trends, technologies, and open research problems. IEEE Netw..

[7] Huang C.-Y., Hsu C.-H., Chang Y.-C., Chen K.-T. GamingAnywhere: An open cloud gaming system. Proceedings of the 4th ACM Multimedia Systems Conference.

[8] Huang C.-Y., Chen K.-T., Chen D.-Y., Hsu H.-J., Hsu C.-H. (2014). GamingAnywhere: The first open source cloud gaming system. ACM Trans. Multimed. Comput. Commun. Appl. (TOMM).

[9] NVIDIA GeForce Now. https://www.nvidia.com/geforce-now/.

[10] Di Domenico A., Perna G., Trevisan M., Vassio L., Giordano D. (2021). A network analysis on cloud gaming: Stadia, geforce now and psnow. Network.

[11] Suznjevic M., Slivar I., Skorin-Kapov L. Analysis and QoE evaluation of cloud gaming service adaptation under different network conditions: The case of NVIDIA GeForce NOW. Proceedings of the 2016 Eighth International Conference on Quality of Multimedia Experience (QoMEX).

[12] Virtual Desktop. https://www.vrdesktop.net/.

[13] ALVR (Air Light VR). https://github.com/alvr-org/ALVR.

[14] NVIDIA CloudXR Suite. https://developer.nvidia.com/cloudxr-sdk.

[15] Kim H., Kwon Y., Lim H., Kim J., Kim Y., Yeo W. (2021). Recent advances in wearable sensors and integrated functional devices for virtual and augmented reality applications. Adv. Funct. Mater..

[16] Rosu A.G., Simiscuka A.A., Togou M.A., Muntean G.-M. BeTwin: Enhancing VR Experiences with BLE Beacon-Based Digital Twins. Proceedings of the ICC 2023-IEEE International Conference on Communications.

[17] Mancuso V., Borghesi F., Chirico A., Bruni F., Sarcinella E.D., Pedroli E., Cipresso P. (2024). IAVRS—International Affective Virtual Reality System: Psychometric Assessment of 360° Images by Using Psychophysiological Data. Sensors.

[18] Shin R., Choi B., Choi S.-M., Lee S. (2024). Implementation and evaluation of walk-in-place using a low-cost motion-capture device for virtual reality applications. Sensors.

[19] Kulozik J., Jarrassé N. (2024). Evaluating the precision of the HTC VIVE Ultimate Tracker with robotic and human movements under varied environmental conditions. arXiv.

[20] Queck D., Albert I., Burkard N., Zimmer P., Volkmar G., Dänekas B., Malaka R., Herrlich M. SpiderClip: Towards an open source system for wearable device simulation in virtual reality. Proceedings of the CHI Conference on Human Factors in Computing Systems Extended Abstracts.

[21] Unity Render Streaming. https://github.com/Unity-Technologies/UnityRenderStreaming.

[22] Petrangeli S., Pauwels D., van der Hooft J., Wauters T., De Turck F., Slowack J. Improving quality and scalability of WebRTC video collaboration applications. Proceedings of the 9th ACM Multimedia Systems Conference.

[23] Gunkel S.N., Hindriks R., Assal K.M.E., Stokking H.M., Dijkstra-Soudarissanane S., Haar F.T., Niamut O. VRComm: An end-to-end web system for real-time photorealistic social VR communication. Proceedings of the 12th ACM Multimedia Systems Conference.

[24] Uhrina M., Sevcik L., Bienik J., Smatanova L. (2024). Performance Comparison of VVC, AV1, HEVC, and AVC for High Resolutions. Electronics.

[25] Han J., Li B., Mukherjee D., Chiang C.-H., Grange A., Chen C., Su H., Parker S., Deng S., Joshi U. (2021). A technical overview of AV1. Proc. IEEE.

[26] Garcia B., Gortazar F., Lopez-Fernandez L., Gallego M., Paris M. (2017). WebRTC testing: Challenges and practical solutions. IEEE Commun. Stand. Mag..

[27] Illahi G., Siekkinen M., Masala E. Foveated video streaming for cloud gaming. Proceedings of the 2017 IEEE 19th International Workshop on Multimedia Signal Processing (MMSP).

[28] Hsiao L., Krajancich B., Levis P., Wetzstein G., Winstein K. (2022). Towards retina-quality VR video streaming: 15ms could save you 80% of your bandwidth. ACM SIGCOMM Comput. Commun. Rev..

[29] Yu H., Shokrnezhad M., Taleb T., Li R., Song J. (2023). Toward 6g-based metaverse: Supporting highly-dynamic deterministic multi-user extended reality services. IEEE Netw..

